# Mesoporous Iron(III)-Doped Hydroxyapatite Nanopowders Obtained via Iron Oxalate

**DOI:** 10.3390/nano11030811

**Published:** 2021-03-22

**Authors:** Margarita A. Goldberg, Marat R. Gafurov, Fadis F. Murzakhanov, Alexander S. Fomin, Olga S. Antonova, Dinara R. Khairutdinova, Andrew V. Pyataev, Olga N. Makshakova, Anatoliy A. Konovalov, Alexander V. Leonov, Suraya A. Akhmedova, Irina K. Sviridova, Natalia S. Sergeeva, Sergey M. Barinov, Vladimir S. Komlev

**Affiliations:** 1 A.A. Baikov Institute of Metallurgy and Materials Science, Russian Academy of Sciences, Moscow 119334, Russia; afomin@imet.ac.ru (A.S.F.); oantonova@imet.ac.ru (O.S.A.); dvdr@list.ru (D.R.K.); ak357@rambler.ru (A.A.K.); barinov_s@mail.ru (S.M.B.); komlev@mail.ru (V.S.K.); 2 Institute of Physics, Kazan Federal University, 18 Kremlevskaya Str., Kazan 420008, Russia; murzakhanov.fadis@yandex.ru (F.F.M.); 151Eu@mail.ru (A.V.P.); 3 FRC Kazan Scientific Center of Russian Academy of Sciences, Kazan Institute of Biochemistry and Biophysics, Kazan 420111, Russia; olga.makshakova@kibb.knc.ru; 4 Department of Chemistry, M.V. Lomonosov Moscow State University, Moscow 119991, Russia; avleonov49@gmail.com; 5 National Medical Research Radiological Center of the Ministry of Health of the Russian Federation, Moscow 125284, Russia; 89060431777@mail.ru (S.A.A.); firelife@mail.ru (I.K.S.); prognoz.06@mail.ru (N.S.S.)

**Keywords:** hydroxyapatite, synthesis, Fe-substitution, mesoporous powder, surface area, iron oxalate, electron paramagnetic resonance, Mössbauer spectroscopy, Density Functional Theory, in vitro investigations

## Abstract

Mesoporous hydroxyapatite (HA) and iron(III)-doped HA (Fe-HA) are attractive materials for biomedical, catalytic, and environmental applications. In the present study, the nanopowders of HA and Fe-HA with a specific surface area up to 194.5 m^2^/g were synthesized by a simple precipitation route using iron oxalate as a source of Fe^3+^ cations. The influence of Fe^3+^ amount on the phase composition, powders morphology, Brunauer–Emmett–Teller (BET) specific surface area (S), and pore size distribution were investigated, as well as electron paramagnetic resonance and Mössbauer spectroscopy analysis were performed. According to obtained data, the Fe^3+^ ions were incorporated in the HA lattice, and also amorphous Fe oxides were formed contributed to the gradual increase in the S and pore volume of the powders. The Density Functional Theory calculations supported these findings and revealed Fe^3+^ inclusion in the crystalline region with the hybridization among Fe-3d and O-2p orbitals and a partly covalent bond formation, whilst the inclusion of Fe oxides assumed crystallinity damage and rather occurred in amorphous regions of HA nanomaterial. In vitro tests based on the MG-63 cell line demonstrated that the introduction of Fe^3+^ does not cause cytotoxicity and led to the enhanced cytocompatibility of HA.

## 1. Introduction

Hydroxyapatite (HA) Ca_10_(PO_4_)_6_(OH)_2_ is the main inorganic compound of the bone tissue and has great importance in the biomedical field as a material for implantation and coatings [1,2]. At the same time, a HA is applied in the areas where morphology, porosity, and BET specific surface area (S) of the nanopowders play a significant role [3]. The importance of the catalytic, adsorption, and drug delivery fields increase rapidly in the last decades [4]. The synthesis of mesoporous HA became the focus of the investigators. Mesoporous HA powders were synthesized by different routes, for example hydrothermal [5,6], sol-gel [7], template-based [8], mechanochemical processes [9], self-assembly including based on pore expander approaches [10,11]. To obtain a highly dispersed HA, one of the most widely used simple and low-cost methods is the precipitation from aqueous solutions containing Ca^2+^ and PO_4_^3−^ ions [12]. Typical precursors are calcium nitrate and di-substituted ammonium hydrogen phosphate [13]; ammonia is used to maintain a constant pH value. To obtain powder materials with various properties (phase composition, morphology, etc.), the authors vary the synthesis conditions: temperature and pH values [14], the concentration of the initial aqueous solutions [15], as well as the Ca–P ratio [14]. It was shown that increasing the synthesis temperature to 90 °C reduces the solubility of HA and, consequently, the loss of Ca, which leads to the formation of 100% HA of materials, while contributing to an increase in the S from 92 (at 25 °C) to 154 m^2^/g (at 90 °С) at pH value 11. The use of surfactants (L-asparagine) to increase the dispersion of powder materials, as well as the introduction of zinc ions, increased the S from 88 to 136 m^2^/g [16]. In [17], a microwave-assistance method led to obtaining a HA powder in the form of rods with an average particle length of about 110 ± 15 nm. Additionally, in [18], HA powder with a particle size of 20–40 nm was obtained using the sol-gel method. Our investigations previously demonstrated an increase in the S of composite materials in the HA-calcium carbonate system, which made it possible to obtain powders with a specific surface of more than 200 m^2^/g with the introduction of 20 wt.% calcium carbonate and the use of the ripening method in the mother liquor [19]. The same approach was applied when the Al-substituted HA powders were synthesized and the powders with 40–60 nm and S up to 91 m^2^/g were obtained [20,21].

At the same time, iron-dopped HA, as well as composite materials based on Fe_3_O_4_ and HA, attract attention due to the special features of the materials. Fe-HA and Fe oxide nanoparticles demonstrated high efficiency as a catalyst in the field of heavy oil purification via oxidative desulfurization [22] and aquathermolysis [23,24] methods. For biomedical application introduction of Fe^3+^ improved bactericidal and mineralizing properties of nanosized HA [25] and also demonstrated a positive impact on the osteoblast-like behavior [26]. Fe-HA powders are promising for cancer monitoring [27], as well as magnetic resonance imaging [28], drug delivery, and heat mediation for the hyperthermia treatment of cancers [29]. Although the influence of Fe^3+^ content on the physicochemical properties including crystalline size was discussed previously [30], there is no data on the formation of porosity and dependence of Fe^3+^ amount on the S of the powders. The introduction of Fe_3_O_4_ nanoparticles increased the S [31] up to 124 m^2^/g compared to 116 m^2^/g demonstrated for pure HA and also up to 148 m^2^/g HA-coated Fe_3_O_4_ compared to 141 m^2^/g for pure HA [32], these developed materials demonstrated controlled drug release of the doxorubicin. In our paper, we have synthesized and investigated iron-doped HA nanopowders and demonstrated as high S as 194.5 m^2^/g for 10 mol. % of Fe^3+^ content. In addition, we performed electron paramagnetic resonance (EPR) and Mössbauer spectroscopy investigations to establish the iron valency and its crystal environment in the final products. Calculations for the structural positions of Fe^3+^ ions based on the Density Functional Theory (DFT) were performed. Cytotoxicity and cytocompatibility in vitro tests were conducted.

## 2. Materials and Methods

### 2.1. Powders Synthesis

Powder synthesis was carried out according to reaction 1 via precipitation method using reactants of analytical grade and deionized water.

(10 − 3x/2)Ca(NO_3_)_2_ + 6(NH_4_)_2_HPO_4_ + (x/2)Fe_2_(C_2_О_4_)_3_ + 8NH_4_OH^–^ > Са_(10−3х/2)_Fe_x_(PO_4_)_6_(OH)_2_ + 20NH_4_NO_3_ + 6H_2_O + 3х/2CO_2_ (1)

х = 0.0; 0.005; 0.025; 0.05; 0.25; 0.5; 1.0.

Calcium nitrate solution and ferrous oxalate solution were mixed with a diammonium hydrogen phosphate solution in selected ratios. The ferrous oxalate amount was taken with slight excess (5 wt.% to stoichiometric one) for the security of full introduction of predicted Fe concentration. The pH value of the reaction mixture was maintained at a level of 12.0–12.5 by adding 25% aqueous ammonia solution. Powders were ripened at mother solution for 21 days at a temperature of 25 °C for full crystallization of precipitate [19]. Obtained powders were filtered, washed in distilled water by triple times, and dried at 60 °C for 24 h. 

### 2.2. Powders Characterizations

For determination of Fe content in the precipitates, powders were heat-treated at 600 °С for water removing, dissolved in HCl–HNO_3_ mixture, and analyzed by atomic emission spectrometry with inductively coupled plasma (AES-ICP, Vista Pro). The phase composition of the powders was characterized by the X-ray diffraction (XRD) method (Shimadzu XRD-6000, CuKα radiation) with identification according to JCPDS and PCPDFWIN databases. Quantitative phase analysis and lattice parameter estimation based on the Rietveld method, as well as a calculation of crystalline size (D) according to Scherrer equation were performed applied the PHAN% software [33]. 

Fourier-transform infrared spectroscopy (FTIR) absorption spectra of the synthesized powders were recorded in the range from 4000 to 400 cm^−1^ using the KBr method on a Thermo Nicolet Avatar 330 FTIR spectrometer.

For the microscopy measurements, the samples were dispersed in ethanol, crushed in an agate mortar, and deposited on a carbon-coated copper grid for transmission electron microscopy (TEM) and high angle annular dark-field scanning transmission electron microscopy (HAADF-STEM) measurements. TEM images and STEM-images were acquired using a Titan 80–300 (FEI, Hillsboro, OR, USA) electron microscope operated at 300 kV. 

Scanning electron microscopy (SEM) images were taken with Helios NanoLab 600i dual beamfocused ion beam (FIB)/SEM microscope (Thermo Fisher, Waltham, MA, USA) and Tescan Vega II with X-ray energy dispersive spectroscopy (EDS) (EDX, Inca, Oxford Instruments, Abingdon, UK).

The S according to Brunauer, Emmett, and Teller (BET), pore volume and pore size distributions were determined using the Barrett–Joyner–Halenda (BJH) analyzer model (Barrett, Joyner, and Halenda) of the materials were determined by low-temperature nitrogen adsorption measurements (Micromeritics TriStar analyzer).

Continuous-wave (CW) EPR spectra of the samples were obtained using Bruker Elexsys E580 spectrometer operating at 9.4–9.9 GHz (X-band) microwave frequency at room temperature. All parameters of setup for CW mode are chosen to avoid distortion, saturation, or overmodulation of the recorded EPR signal (amplitude of modulation M = 5 G and mw power P = 2 μW).

The Mössbauer spectra were obtained on the MS-1104 Em spectrometer in the continuous acceleration mode at room temperature using a symmetric sawtooth law of velocity change with separate accumulation of the spectra as the source moves forward and backward and their subsequent summation to eliminate background line distortion. A scintillation counter with a thin NaJ(Tl) crystal was used as a detector. The spectra were obtained with a ^57^Co source in the Rh matrix. Calibration was performed using the α-Fe spectrum, and isomer shifts were measured from the “center of gravity” of the spectrum obtained at room temperature of this reference. Mathematical handling of the gained spectra was carried out through the standard Mössbauer program UnivemMS.

### 2.3. DFT Calculations

The calculations were performed on the 1 × 1 × 1 monoclinic HA (88 atoms in the cell) using initial coordinates from [34]. Such a system was proved to be relevant for the reproduction spectral parameters of dopped HA crystals [35]. The cell geometry optimization was performed based on Density Functional Theory with the plane-wave basis and Vanderbilt ultrasoft pseudopotentials [36] using the Quantum ESPRESSO program package [37]. The generalized-gradient approximation for the exchange-correlation functional of Perdew, Burke, and Ernzerhof (GGA-PBE) [38] was used. The kinetic energy cutoffs of 45 Ry for the smooth part of the electron wave functions and 300 Ry for the augmented electron density were set up (in agreement with previously denoted [21]). Both atomic positions and lattice parameters were allowed to relax in the course of geometry optimization. The convergence condition on forces was 10^−3^ a.u. The Brillouin Zone integration was performed on a Monkhorst-Pack 2 × 2 × 1 k-point mesh [39]. The electron structure was calculated using the refined structure under the gauge-including projector augmented wave method with the Troullier−Martins norm-conserving pseudopotentials [40] and a plane-wave energy cutoff of 70 Ry.

The spin-polarized calculations were performed for iron-doped HA. For the charge compensation, the following scheme was used Ca_10−x_(PO_4_)_6_(OH)_2−x_, where x = 1. This implies the vacancy formation at hydroxyl upon Ca^2+^ substitution by Fe^3+^. The scheme was previously used by Jiang et al. [41]

### 2.4. Cytocompatibility Test

To evaluate the potential biomedical application of the synthesized powders, in vitro investigations for determination of cytotoxicity and cytocompatibility were carried out on the sintered at 1200 °C granules based on HA and Fe-HA. The bioceramic granules are a well-known form of osteoconductive material for orthopedics and dentistry. Sintered granules were obtained in accordance with the developed in our laboratory technology described by Komlev et al. [42]. Shortly, the granules were formed due to the effect of immiscible liquids of the powders-contained hot gelatin-based slurry and cold oil media during the rotation of an overhead stirrer, wash-out with acetone, air-dried, and sintered at 1200 °C for 2 h in the air atmosphere. The granules were classified with caprone sieves to obtain the desired fraction of 300–600 µm. The sterilization was performed by dry heating at 180 °С for 1.5 h in Binder, USA. The cytotoxicity investigations were carried out in accordance with ISO 10993.5-99 by direct contact of the extract (0.2 g of material in 1 mL of complete growth medium (CGM) that contained DMEM medium (PanEko, Moscow, Russia), 10% fetal bovine serum (PAA, Coelbe, Germany), glutamine (0.65 mg/mL, PanEko, Russia), and gentamycin (50 μg/mL, PanEko, Russia) with the test culture—cell line of human osteosarcoma MG-63 (Russian Collection of Сell Cultures, Institute of Cytology, Russian Academy of Sciences, St. Petersburg, Russia). Extraction was carried out for 24 h at a temperature of 37 °C with constant stirring on an orbital shaker (Elmi, Rīga, Latvia). The pH values of the solution were determined for each sample of extracts (Hanna, Rhode Island, USA). To assess the possible toxicity of the extracts of the obtained bioceramic granules, after 24 h of incubation MG-63 growth medium was taken from the wells with the test culture and 200 μL of the obtained extracts wеre replaced. As a control, pure CGM was used.

The viability of the MG-63 culture was determined after 24 h using the MTT test [43]. The optical density of formazan, solution, which is reflected in the mitochondrial activity of viable cells from MTT, was measured at 540 nm using a Multiskan FC microplate photometer (Thermoscientific, Waltham, MA, USA).

For each sample extracts, the toxicity index (TI) was calculated according to ISO 10993.5-99 by the Equation (2):

TI = 100% − OD exp/ODcontrol (%),(2)

where OD is the optical density of the formazan solution in the experiment and the control, respectively, and their ratio essentially represents a pool of viable cells (PVC). A sample of the material was considered non-toxic when an IT value is lower than 30%.

The cytocompatibility was investigated during the direct contact of cells with ceramic materials. Bioceramic granules were placed into 96 well plates for cultivation (Corning Costar, Cambridge, MA, USA) into triplets with one plate per each incubation period and covered with complete growth medium CGM. After the establishment of the neutral values of pH, the plates with examined samples and control with (cultural plastic, polystyrene) were introduced with a cell suspension (the MG-63 culture at a density of 7000 cells per well) at 200 μL of the CGM, which was incubated for 1, 3, and 7 days with regular replacements of the CGM. All the procedures were performed under sterile conditions in an atmosphere of moist air that contained 5% CO_2_ at 37 °C. The viability of MG-63 cells over time was measured with the MTT test. The pool of viable cells (PVC) was evaluated at different times of the experiment. A specimen was assumed to be cytocompatible in the absence of cytotoxicity (PVC ≥ 70%) on a certain day of cell growth.

The obtained results were processed by conventional methods of variational statistics using Microsoft Excel 2000. The significance of differences was assessed using a parametric Student t-test; differences were considered statistically significant at *p* < 0.05.

## 3. Results

### 3.1. Powders Chemical and Phase Composition

According to AES-ICPdata, the chemical composition of the powders was close to the predicted ones (Table 1).

**Figure 1:**
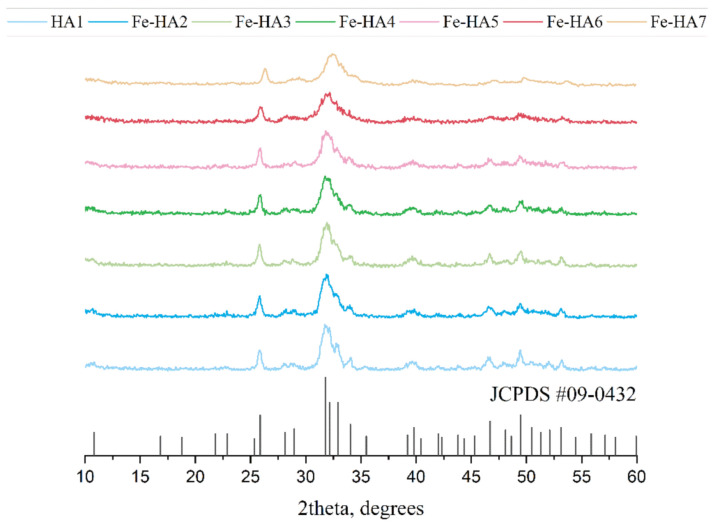
XRD spectra of hydroxyapatite (HA) and Fe-HA powders after the synthesis, JCPDS#09-0432 corresponds to HA.

According to XRD analysis, all powders were presented by apatite structure (JCPDS No: 9-432) with a low crystallinity degree (Figure 1). As the amount of Fe increase from HA to Fe-HA7, the resolution decreased and broadening of the peaks was observed. The calculation of D revealed the gradual fall of the crystalline sizes from 13.8 (0.3) nm to 7.5 (0.6) nm (Table 2). There were no additional peaks of any Fe oxide phases were detected. The lattice parameters slightly decreased as the Fe^3+^ was introduced due to the difference in the ionic radii of iron (0.67 Å) compared to calcium (1.04 Å), but the low degree of the crystallinity made these changes inconspicuous. There was also a tendency of c/a ratio decrease with a higher amount of the Fe^3+^ which will contribute to the morphology of the powders.

### 3.2. FTIR Investigation

**Figure 2:**
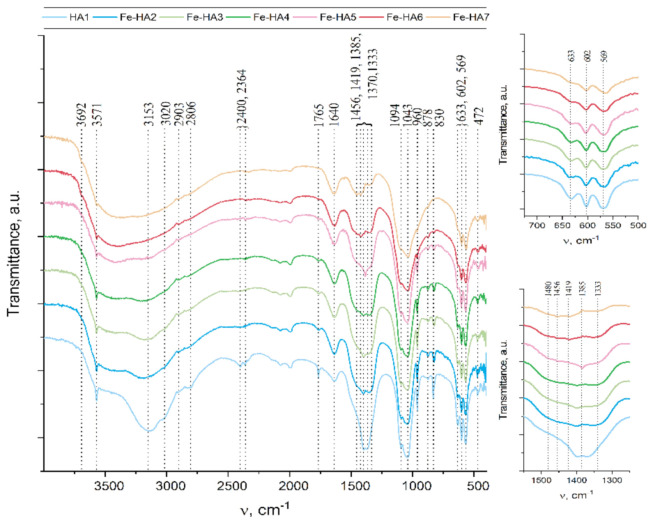
FTIR data of the Fe-HA powders.

The main phosphate asymmetric stretching vibration mode ν_3_ appears at 1094 and 1043 cm^−1^, so as ν_1_ symmetric stretching vibration mode at 960 cm^−1^ (Figure 2) Additionally, ν_4_ O-P-O bend is evidently appears at 602 and 559 cm^−1^ [44] and double generating bending mode (ν_2_) PO^4^ at 472 cm^−1^ [45]. Additionally, there were peaks associated with fluctuations group HPO_4_ at 878 cm^−1^ [46]. It should be noted that the intensity and resolution of these peaks significantly decrease with the Fe^3+^ content increase. Thus, for Fe-HA7 sample HPO_4_ peak at 878 cm^−1^ detected as a shoulder in Fe-HA7 spectra.

Another important characteristic feature of the apatite spectra is the oscillations associated with the hydroxyl group. Such bands at 3571 cm^−1^ and 633 cm^−1^ appear for all samples, but their intensity significantly decreases with the growth of Fe^3+^ content, so as in Fe-HA7 sample peak at 633 cm^−1^ detected as a shoulder. A decrease in the intensity of the hydroxyl group vibrations is associated with a decrease in the degree of crystallinity of the apatite structure owing to the nonstoichiometric substitution of Fe(III) for Ca(II) [45]. It should be noted that at high Fe^3+^ content additional band at 3692 cm^−1^ appear. It may be concerned with non-apatite hydroxyl groups. Thus, in [47] this peak was associated with NaOH hydroxyls.

The presence of the H_2_O bending mode at 1640 cm^−1^ so as a broad halo in the range 2500 to 3500 cm^−1^ indicates the presence of a significant amount of water in all samples [46].

The appearance of the remaining peaks in the spectrum is associated with the presence of CO_2_ from the air (1500–1400 cm^−1^ and at 2400 cm^−1^ regions) [48] and with co-product of the reaction—ammonium nitrate: in the range of 1300 to 1550 cm^−1^, several peaks were corresponding to vibrations of the NO_3_, as well as peaks at 830 and 1765 cm^−1^, and poor-resolved bands at 2800–2900 cm^−1^ for NH_4_ [49].

The question of the introduction of carbonate anions into the apatite structure is of particular interest. As it is well-known, carbonate ions can replace both phosphate groups (B-type) and hydroxyl groups (A-type) in the hydroxyapatite structure [50]. The wide shoulder in the region of 1480 cm^−1^ is attributed to the presence of surface labile carbonate [51]. However, for samples Fe-HA6 and Fe-HA7, distinct peaks are observed at 1456 and 1419 cm^−1^. As indicated in [52], for B-type substitutions, the ν_3_ antisymmetric stretching vibration splits into ν_3a_ and ν_3b_ peaks at 1423 and 1456 cm^−1^, respectively. Meanwhile, there are no peaks that indicate an A-type substitution (1530 and 1465 cm^−1^) [53]. Thus, in the case of Fe-HA6 and Fe-HA7, the presence of not only surface labile carbonate, but also a slight entry of B-type carbonate ions into the apatite structure was detected.

### 3.3. Microscopy Investigations

**Figure 3:**
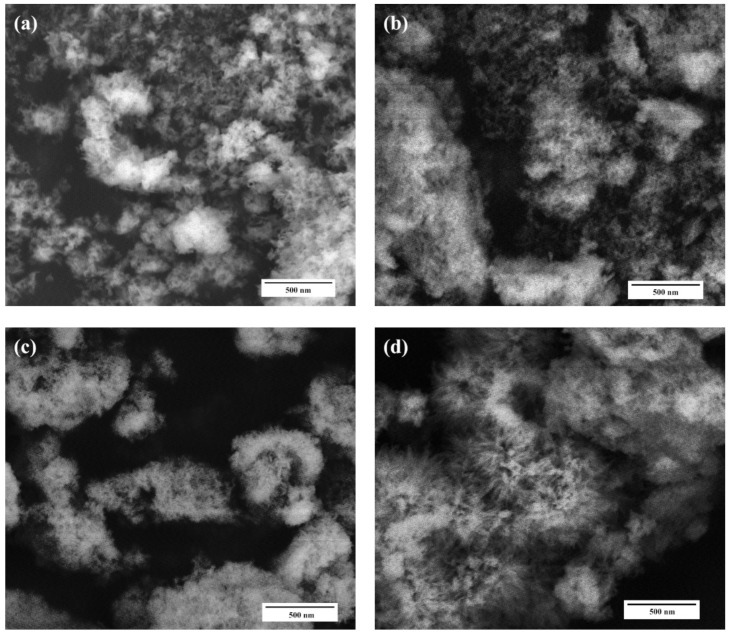
SEM microphotographs of the HA (**a**), Fe-HA3 (**b**), Fe-HA4 (**c**), Fe-HA7 (**d**) samples, magnification 50 kX.

**Figure 4:**
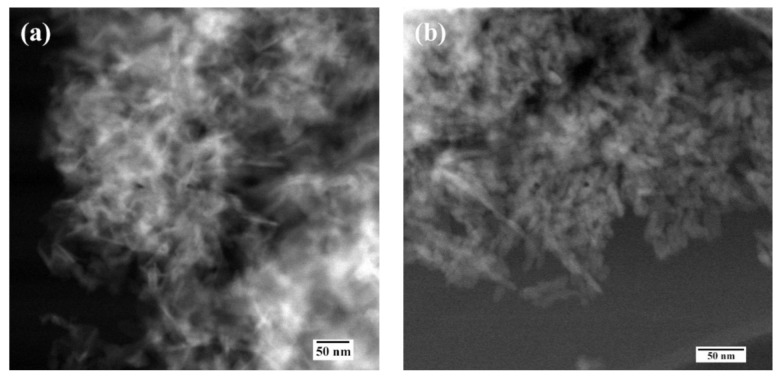

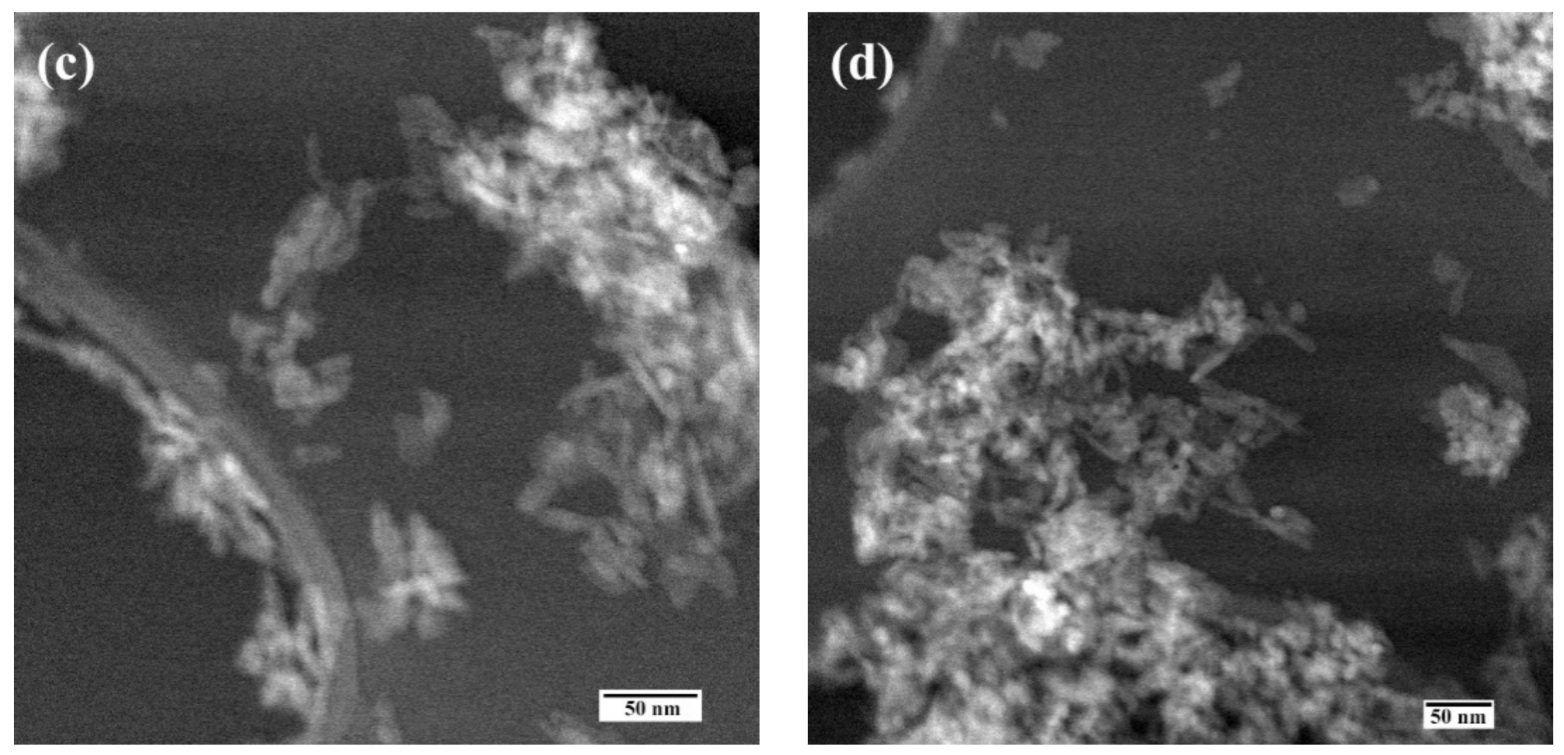
Scanning transmission electron microscopy data of the HA (**a**), Fe-HA3 (**b**), Fe-HA4 (**c**), Fe-HA7 (**d**) powders.

According to SEM data, all powder materials HA–Fe-HA7 observed as plate-like particles previously with hexagonal shapes in the case of pure HA with a tendency to form a needle-like structure with an increase in Fe^3+^ content. The increase in Fe^3+^ content led to a gradual decrease in particle length from 30–100 nm (HA) to 30–50 nm (Fe-HA7), the plate thickness diminished from 10 to 2–3 nm. The decrease in the grain size with an increase in Fe^3+^ content could be due to the smaller ionic radius of iron (0.67 Å) compared to calcium (1.04 Å) and confirmed by data of D according to XRD. HA particles form loose agglomerates and the introduction of Fe^3+^ resulted in a decrease in the density of agglomerates with the increase in Fe^3+^ content (Figure 3). It should be noted, that according to lattice parameters calculation, the increase in Fe^3+^ amount resulted in the growth of the c/a ratio. The STEM data confirmed the tendency to form needle-like and rod-shaped particles with lengths up to 50 nm compared with hexagonal ones for pure HA (Figure 4). Previously, in [30] the increase in Fe^3+^ amount resulted in the formation of the spherical structures compared to rod-like for pure HA. Fe-HA synthesized from Ca(OH)_2_ and FeCl_3_ as sources of the cations resulted in the formation of acicular particles of irregular contour with a size of 50–100 nm [54]. The round-shaped nanoparticles with a size below 100 nm were obtained by Ribeiro et al. [27]. In the case of Fe^2+,^ the formation of nanorods-like mesoporous powders was achieved by precipitation with further autoclave treatment [55]. In our investigation, the aggregates of the roads and needle-like particles induced the formation of the mesopores among them, and an increase in Fe^3+^ content resulted in the broadening in the pore size distribution as will be presented further.

**Figure 5:**
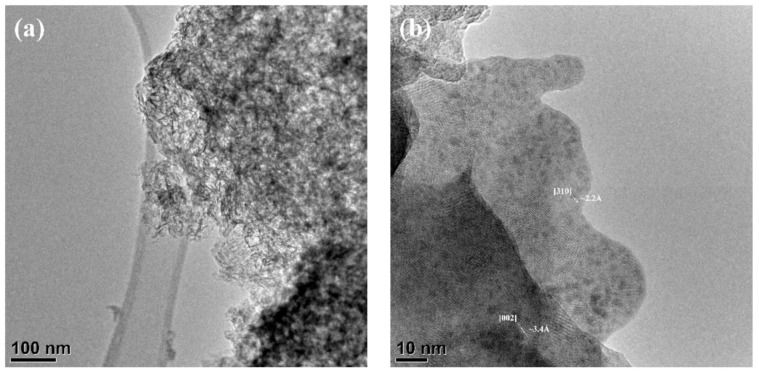
High-resolution TEM data of the Fe-HA7 (**a**,**b**) powders.

According to HR TEM data of HA-Fe7 powder, despite the low crystallinity degree, the crystal structure of the apatite phase was observed and lattice directions of [301] and [002] were evaluated (Figure 5).

### 3.4. BET, Pore-Volume, and Adsorption Average Pore Width and Diameter for the Synthesized Samples

**Figure 6:**
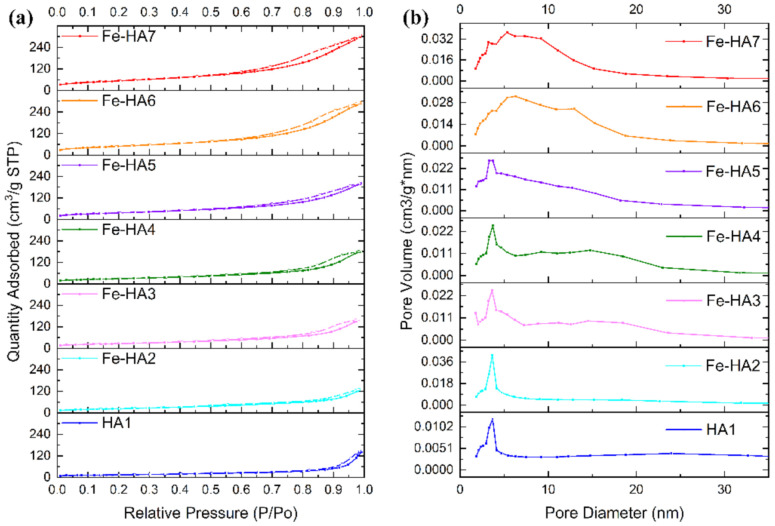
Nitrogen adsorption-desorption isotherms (**a**) and pore size distributions (**b**) of HA and Fe-HA powders.

Nitrogen sorption isotherms for synthesized mesoporous HA and Fe-HA powders are presented in Figure 6 with detailed characteristics outlined in Table 3. According to BET data, all the obtained compositions are mesoporous powders, and the increase in Fe^3+^ content resulted in the gradual growth of the S from 52.1 to 194.5 m^2^/g and total pore volume from 0.16 to 0.43 cm^3^/g in the range of the materials HA1–Fe-HA7. According to Figure 6, the adsorption-desorption isotherm for powders was type IV, while the hysteresis loop showed H3-type behavior illustrating split-shape pores formed by plane-parallel particles confirmed by SEM and STEM data [56,57]. The pores were formed in the space between particles, which size dramatically decrease according to the calculation of D and STEM observations. The pore size distributions computed based on BJH analysis indicated that materials HA–Fe-HA5 had a distribution with a noticeable maximum at 3.6 nm characterized for uniform pore texture. At the same time, powders Fe-HA6–Fe-HA7 demonstrated the expansion of the curves centered around 6.5 nm reflecting the formation of pores with the broader size distribution. As a result, a significant increase in total pore volume up to 0.43 cm^3^/g was presented for materials Fe-HA6–Fe-HA7. The data on mesoporous HA indicates an H3-type hysteresis loop for mesoporous HA, as it was previously demonstrated by [56,58], but S of presented powders were limited by 42 and 35 m^2^/g. At the same time, synthesized mesoporous HA particles characterized by S up to 142 m^2^/g using co-precipitation synthesis with template based on co-polymer pluronic F127d with the H1 hysteresis loop characterized for cylindrical pores with open ends [32,59]. Fe(II)-dopped nanopowders demonstrated an H3-type hysteresis loop [55], but data on Fe(III)-substituted mesoporous HA characteristics is limited to the best of author knowledge up to day. In our work we estimated the increase in the gas adsorption capacity Fe(III)-HA compared to pure HA and did not observe the changes in the type of hysteresis loop with an increase in Fe^3+^ content and corresponding growth of the surface area.

### 3.5. Electron Paramagnetic Resonance Spectroscopy

**Figure 7:**
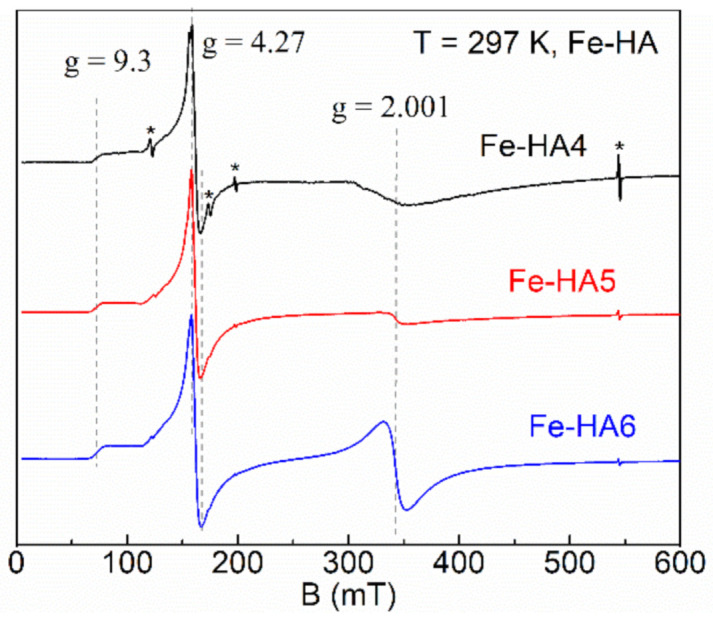
Normalized electron paramagnetic resonance (EPR) spectra for Fe-HA4 (upper curve), Fe-HA5 (middle curve), and Fe-HA6 (lower curve) samples. Dashed lines indicate the absorption features of Fe^3+^ ions; asterisks indicate the internal EPR cavity signals used as references.

The sample of pure hydroxyapatite is EPR silent since there are not any paramagnetic centers (PC). Owing to the electronic configuration of Fe^3+^ ions (3d^5^, ground state ^6^S_5/2_, electronic spin S = 5/2), it became possible to detect EPR signals for the Fe-doped HA. In the spectra (see Figure 7), three isotropic transitions are observed at g = 2.001, g = 4.27 and a shoulder at g = 9.3 for all iron concentrations which are distinctive features of Fe^3+^ ions [60,61].

Conventionally, the iron-containing systems can be described by the following spin-Hamiltonian for isotropic g-tensor [41,62]:

(3) $$H={g\beta B}_{0}S_{z}+D\left(S_{z}^{2}-S\left(S+1\right)/3\right)+E\left(S_{x}^{2}-S_{y}^{2}\right),$$

where β is Bohr magneton, D and E are zero-field splitting parameters (D > 1/3E >> gβB_0_), S_x,y,z_ are the projections of S onto the main axes of g-tensor. The large value of zero-field splitting results in the appearance of low-field resonance spectra features (signal at g = 4.27 with a shoulder up to g = 9.3, Figure 7). Thus, we consider the values of D and E parameters are greater than ≈9.6 GHz (operating frequency at X-band) and respective ratio E = D/3 ≈ 3.2 GHz for this spin system of Fe-HA. As the orbital magnetic moment of Fe^3+^ ions is L = 0, it is expected that the value of the g-factor is close to that for the free electron (g_e_ = 2.0023) [62]. The integral intensity of the spectra is proportional to the amount of the doped iron (Table 1) indicating the success of the Fe-doping procedure of HA with Fe^3+^ ions.

The physical origin of the single line (g = 2.001) is related to the spin transition with ∆M_S_ = ±1 for the lowest Kramers doublet (D > 0). This type of signal is common for the non-isolated ions in octahedral coordination with strong spin–spin interaction coupling. It has been attributed by various authors to separate ferric oxide phases, a variety of structural Fe^3+^ species with overlapping signals, surface Fe oxide or oxyhydroxides, Fe…O...Fe clusters, and adsorbed Fe^3+^ on HA [63,64,65]. In contrast, the asymmetric low-field resonance features originate from the isolated Fe^3+^ ions in a tetragonal environment with rhombic distortion. A noticeable redistribution of the resonance intensities between the signals at g = 4.27 and g = 2.001 (starting at about 0.5–1 mol. % of the iron amount) can be attributed to the additional paramagnetic complex, probably in the form of the iron oxide (Fe_2_O_3_) [66,67].

### 3.6. Mössbauer Spectroscopy

**Figure 8:**
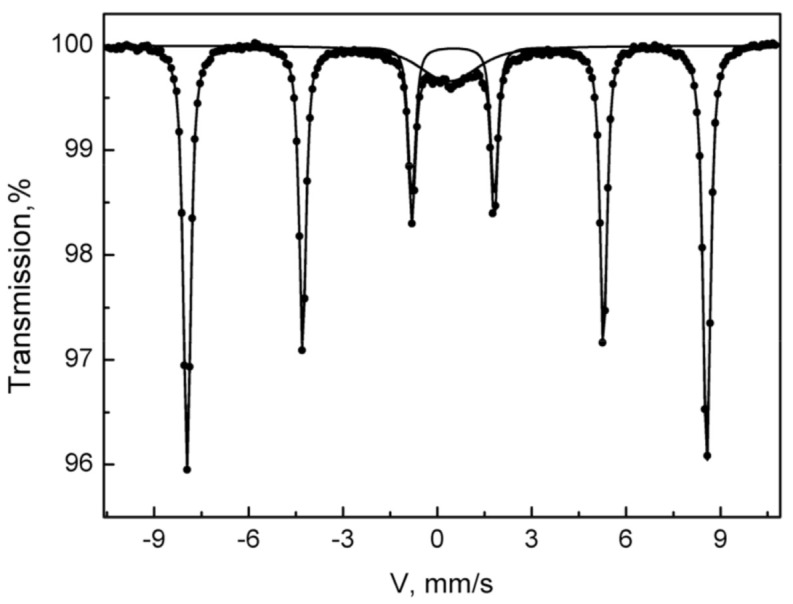
The Mössbauer spectrum of the Fe-HA7 sample was acquired at room temperature.

To prove the presence of the iron-oxides, Mössbauer spectroscopy for some of the synthesized species was applied. The registered Mössbauer spectrum of the sample Fe-HA7 at room temperature is shown in Figure 8. The parameters of mathematical processing are summarized in Table 4. The spectrum consists of a superposition of an unresolved doublet and a sextet. The sextet in terms of the parameters of hyperfine interactions fully corresponds to the α form of Fe_2_O_3_ and composes 87% of the spectrum area. The non-resolved doublet with an isomeric shift of 0.37 in the center of the spectrum corresponds to superparamagnetic α-Fe_2_O_3_ particles with dimensions that do not lead to spontaneous magnetization at room temperature [68,69].

### 3.7. DFT Calculations

#### 3.7.1. Geometry

Fe^3+^ doped HA undergoes a notable local geometry reorganization. Since Fe^3+^ has a smaller radius than Ca^2+^, it shifts to adjacent orthophosphate groups. To retain sufficient coordination, Fe^3+^ ion slightly rotates the PO_4_^3−^ groups and attracts an additional oxygen atom, which could be from OH^−^ group. Indeed, many researches outlined the Ca(2) position located in the anion channel as a preferable one for the inclusion of small cations (see our recent paper and references therein [21]). The Fe^3+^ inclusion leads to the anisotropic lattice contraction. Nevertheless, the overall unit cell parameters alter insignificantly within less than 1%.

**Figure 9:**
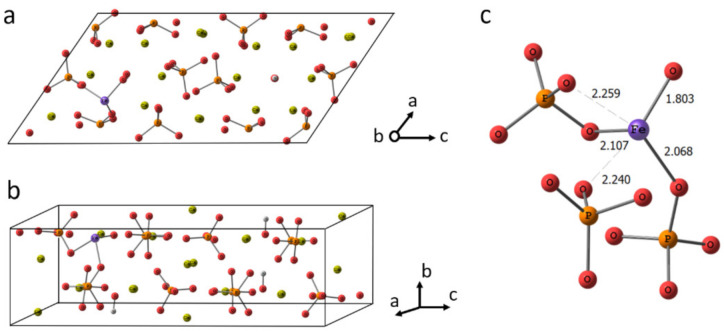
The crystalline cell of HA doped with Fe^3+^ in Ca(2) position (**a**,**b**). The fragment of the cell showing Fe^3+^ coordination (**c**).

In the doped HA optimized in periodic conditions, the Fe^3+^ forms the shortest bond with O from the closest hydroxyl (releasing H^+^), as is shown in Figure 9. Then, two bonds around 2.1 Å in length are formed with the adjacent orthophosphate groups. The other two oxygen atoms occur at a distance around 2.25 Å, which is still smaller than the shortest Ca...O distance amounted to 2.35 Å. The rest of the oxygen atoms are remote more than 3 Å from Fe^3+^. Thus, five atoms in the coordination sphere can be counted. It should be noted the elongation of the P-O bonds providing their oxygen for Fe^3+^ coordination is accompanied by the shortening of another bond in the orthophosphate. That points to the electron density re-distribution upon Fe^3+^ inclusion.

#### 3.7.2. Electron Structure

**Figure 10:**
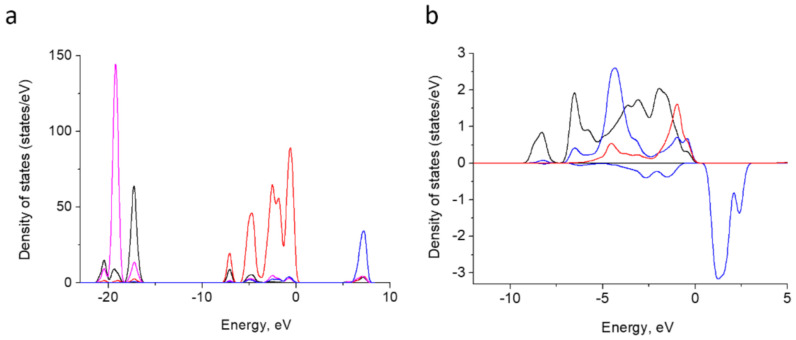
Atom-projected partial density of states curves for Ca-3p (magenta), Ca-3d blue, O-2s (black) and O-2p (red) in HA summed up overall relative atoms (**a**). Atom-projected PDOS curves for Fe-3d (blue; positive values for spin-up and negative ones for spin-down) and O-2p for adjacent oxygen atoms P-linked oxygen atoms (black) and H-vacancy bearing oxygen (red) (**b**).

To provide a deeper insight into the electronic structure of doped HA, the density of states for both pure HA and Fe^3+^ containing HA were calculated. The results for pure HA (Figure 10a) agree well with the previous reports [70,71]. The lower valence bands of HA comprise O-2s and Ca-3p orbitals. The upper valence band is due to O-2p orbitals. The conduction band is organized by Ca-3d and 4s orbitals. The band gap calculated amounts to 5.3 eV.

In the doped HA, Fe-3d band contributes to the upper valence band. It is split into 5 components and locates within 0 to −10 eV (Figure 10b). The bands overlap with O-2p orbitals of O^2−^ and four neighbor oxygen atoms linked to phosphorus. These indicate a hybridization among Fe-3d and O-2p and a partly covalent Fe–O bond formation. This is in agreement with the assumption made on the basis of partial charge estimation [41].

The occupied Fe 3d region in our system displays narrower bands than the fourfold coordinated Fe^3+^ calculated under the embedded cluster approach in [41] and assumes a higher symmetry of the former. The exchange splitting between spin-up and spin-down bands is of ~5 eV.

**Figure 11:**
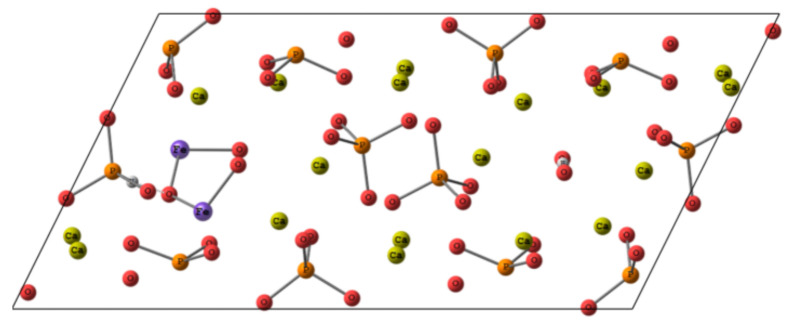
The scheme of putative Fe_2_O_3_ location in HA cell.

Thus, being inserted into Ca(2) position of HA, Fe^3+^ tends to establish close contacts to adjacent oxygen atoms with an onset of covalent bond formation. Therefore, we suppose that the inclusion of Fe_2_O_3_ could also occur in the anion channel upon substitution of two calcium atoms according to the scheme represented in Figure 11. The charge compensation scheme was suggested as following, one PO_4_^3−^ was substituted by PO_3_H^2−^ together with vacancies formation for one proton and two OH^−^ groups. In the conditions of the periodic cell, the convergence of such a system was not achieved. Along with the fact that Fe-O distances in Fe_2_O_3_ remained quite short, within the range of 1.7 to 1.9 Å, the new contacts for iron coordination also tended to shorten. We assume that such a bond shortening requires a more significant contraction than it could be achieved in the periodic cell. Apparently, the inclusion of Fe oxide in the crystalline HA, if any, requires a more prominent re-organization of the surrounding than Fe^3+^ ions and could lead to a lowering of crystallinity degree and some local pronounces defects formation in the sample.

### 3.8. In Vitro Investigations

#### 3.8.1. Granules Characterization

For the investigation of the potential biomedical application of the developed powders, the bioceramic granules of the several compositions—HA1, Fe-HA5, and Fe-HA7 were obtained. All granules were sintered and characterized by mechanical properties appropriated for manipulation during the surgical treatment. According to XRD data (Figure S1), pure HA granules were predominately formed by high crystallinity HA with a tiny amount of β-tricalcium phosphate (β-TCP) which formation is typical for this temperature [14]. The introduction up to 2.5 mol. % Fe^3+^ resulted in the formation of pure HA phase, a similar stabilization was observed for Al-enriched HA [20]. The formation of a mixture of β-TCP with a small amount (5 wt.%) of Fe_2_O_3_ was observed when 10 mol. % Fe^3+^ was introduced (Fe-HA7).

**Figure 12:**
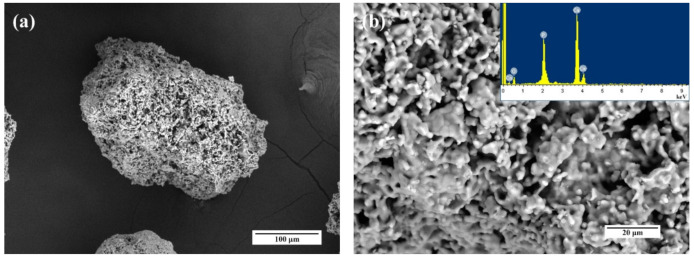

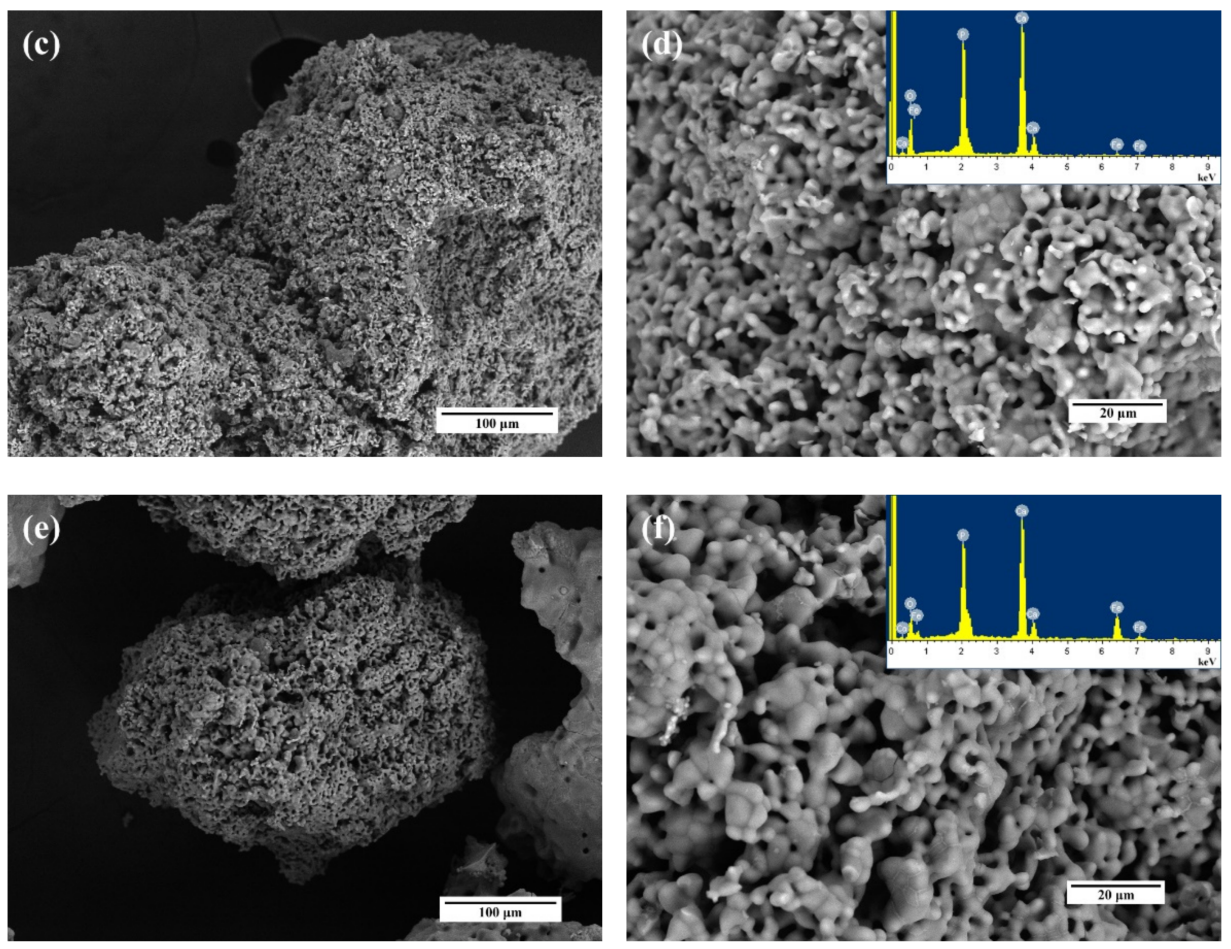
SEM images and EDS spectra of the HA1 (**a**,**b**), Fe-HA5 (**c**,**d**), Fe-HA7 (**e**,**f**) bioceramic granules.

The morphology of the granules was established by SEM (Figure 12). According to obtained data, the granules are characterized by a size of 300–500 µm and the presence of a bimodal porous distribution with 10–20 µm large porous channels and 0.1–0.5 µm small pores.

#### 3.8.2. Cytocompatibility of Iron(III)-Doped HA

According to the MTT test bioceramic granules were non-toxic: after 24 h of incubation of human sarcoma cells with the extracts of HA1, HA-Fe5, HA-Fe7 samples, the size of PVC was close to the control values and amounted to 78.5–98.3%, and the toxicity index was 4.2–21.5% (lower, than 30%), respectively (Table 5). The pH values of the extracts demonstrated a neutral reaction (7.3–7.8).

**Figure 13:**
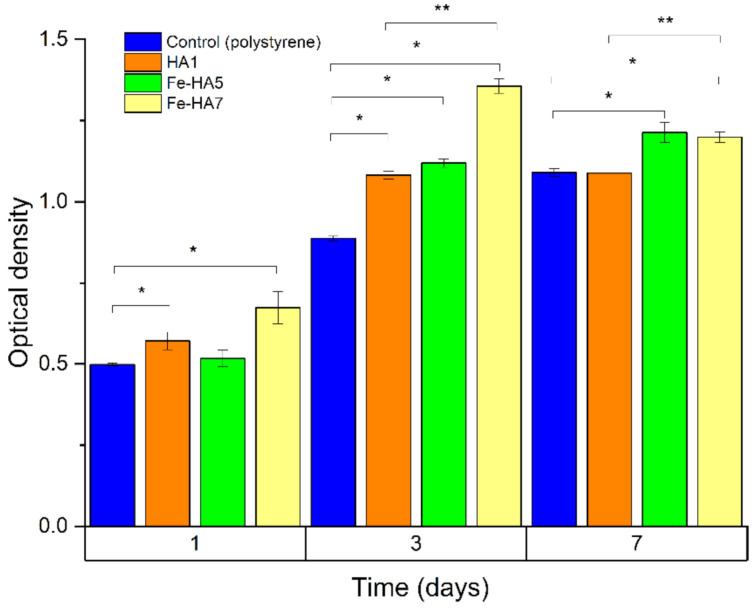
The optical density of formazan solution (OD; a.u., MTT test) in the dynamics of cultivation of human osteosarcoma MG-63 on the bioceramic granules and polystyrene (control). * statistically significant difference between the groups (HA, Fe-HA5, Fe-HA7) and the control (polystyrene). ** statistically significant difference between the groups (Fe-HA5, Fe-HA7) and HA.

According to the MTT test, the obtained granules surfaces supported the adherence and proliferative activity of human MG-63 osteosarcoma cells. In Figure 13, we showed a statistically significant difference between the groups at different days of cell growth. The figure shows that HA-ceramics doped with iron ions are cytocompatible: a statistically significant difference was obtained between the populations of MG 63 cells in the Fe-HA5 and Fe-HA7 groups on the 3rd and 7th days compared to the control (polystyrene) and at the same time between Fe-HA7 and HA. Granules of Fe-HA7 have indicated strongly marked matrix characteristics. Similar behavior of Fe-dopped HA was previously observed by S. Balakrishnan et al. [30] and was linked with a decrease in intracellular reactions due to the fall of free Ca^2+^ ions. Moreover, the biocompatibility of composite powders based on HA and Fe_3_O_4_ nanoparticles was observed in vivo on the New Zealand white rabbits dorsocranial incisions model [72].

This result demonstrates that the introduction of Fe^3+^ did not lead to cytotoxicity and demonstrated cytocompatibility.

## 4. Discussion

In our work, we demonstrated the influence of the Fe^3+^ amount introduction on the phase composition, powder morphology, and mesoporous formation, as well as observed fine structure based EPR and Mössbauer spectroscopy. The synthesis of the mesoporous HA and Fe-HA is a challenge up to date. The application of the organic template is a promising way to increase the pore size volume and the distribution of the pores by the size demonstrated by [9,10], but this method required high-cost equipment and initial reactants. At the same time, the precipitation route is one of the most promising approaches to economically and practically fabricate nano-sized HA powders. Lee et al. synthesized nano-HA by this method and demonstrated the opportunity to increase the S up to 154 m^2^/g based on the control of pH and temperature and Ca/P ratio, but the formation of mesoporous structure and pore volume distribution did not present and discuss in the paper [14]. In our work, we applied the precipitation route at the normal conditions with ripening in mother solution during 21 days and obtained the mesoporous powders characterized by H3 type hysteresis loop. The synthesis of the mesoporous powders led to an increase in the application fields of the materials, for example in the areas of catalysis, water, and earth purification, drug delivery, and biomolecules sorption.

Fe^3+^ ions and Fe oxides play an important role in the biomedical and technical applications of the HA. The synthesis of Fe-dopped HA is typically performed by using Fe(NO_3_)_3_ [30] or FeCl_3_ [45]. At the same time, iron (III) oxalate has good solubility in water and has been demonstrated as a promising precursor to obtain nanosized particles of iron oxides [73]. Impregnation by the solution of iron (III) oxalate of mesoporous silica (SBA-15) material with further heating resulted in the formation of XRD amorphous small oxide particles with a size of 5 nm [74]. In our work, we use iron (III) oxalate as a source of the Fe^3+^ ions. Despite of the absence of the noticeable changes in the lattice parameters detected by XRD, the introduction of the Fe^3+^ in the lattice was confirmed by EPR. EPR is an efficient approach to purity check at very low concentration and quantitative analysis of the doped CaP materials [75]. Simultaneously, the formation of a minor quantity of the Fe oxide was estimated by EPR and was confirmed by Mössbauer spectroscopy when the amount of Fe^3+^ was 10 mol. %. As the XRD data of the synthesized powders did not demonstrate the presence of Fe oxides, we could assume that it was linked with the amorphous stage of nanoparticles due to iron(III) oxalate initial reactant [73] and the minor oxide amount in the samples. This also agrees well with the results of DFT calculations, which showed that the incorporation of Fe^3+^ ion may occur in the crystalline HA since it demands only a minor lattice distortion. In contrast, the inclusion of Fe oxide molecules hardly can happen in a highly crystalline region. Therefore, despite the Fe^3+^ revealed a tendency for a covalent linkage formation with adjacent oxygen atoms, the Fe oxide particles rather occupy amorphous regions in HA nanomaterial.

The introduction of the Fe^3+^ led to a significant increase in the S and pore volume of the mesoporous Fe-HA powders. Similar behavior was demonstrated for Fe(II)-dopped HA, when the introduction of 10 wt.% of Fe^2+^ ions resulted in the increase in the S from 21 (pure HA) to 159 m^2^/g [55]. In our paper, we reached the value of the S as high as 194.5 m^2^/g. The growth of the S is linked with the formation of a needle and plate-like particles with increased compared to pure HA *c/a* ratio and decreased D due to introduction of Fe^3+^. Formation of mesoporous structure with hysteresis loop type H3 is characterized for these particle types [76,77].

The mesoporous particles characterized increased sintering ability due to high surface energy [78]. We observed the transformation of apatite structure in the β-TCP when a high amount of Fe^3+^ was introduced correlated with our previous investigations of the Al^3+^ concentration influence on the thermal stability [20]. Crystallization of the Fe_2_O_3_ was also detected and confirmed our assumption of Fe oxides present in the amorphous stage in the mesoporous powders after the synthesis. We conducted in vitro investigations and confirmed cytocompatibility of the Fe^3+^-doped HA, β-TCP, and Fe-oxide contained composite materials [72,79].

## 5. Conclusions

According to obtained data, the increase in Fe^3+^ content resulted in a significant increase in S and modification of the pore size distribution. In our study, we do not use any template or pore expander and observed significant influence of the only Fe^3+^ incorporation on the S of apatite-structure single-phase mesoporous powders (Fe-HA2–Fe-HA5) up to 134 m^2^/g and also take notice to formation high dispersed composite powders with S up to 194.5 m^2^/g via synthesis by co-precipitation method with ripening in mother solution.

The synthesized powders could be applied as a catalyst, sorption material for water purification, or medical application as a matrix for bone tissue engineering, or as a drug delivery system. EPR spectroscopy confirms the presence of Fe^3+^ ions in the HA structure and jointly with Mössbauer spectroscopy at room temperature shows the formation of iron oxide Fe_2_O_3_ at high iron concentrations of 10 mol. %.

The Density Functional Theory calculations revealed the hybridization among Fe-3d and O-2p orbitals with a partly covalent bond formation upon Fe^3+^ inclusion, whilst the inclusion of Fe oxides assumes crystallinity damage and rather occurs in amorphous regions of HA nanomaterial as concluded from the experiment.

## Figures and Tables

**Table 1:** Powders chemical theoretical and experimental compositions.

Sample Code	Chemical Formula (Theoretical)	Chemical Formula (Experimental)	Fe Concentration, mol. %
HA1	Ca_10_(PO_4_)_6_(OH)_2_	Ca_10_(PO_4_)_6_(OH)_2_	0
Fe-HA2	Ca_9.9925_Fe_0.005_(PO_4_)_6_(OH)_2_	Ca_9.991_Fe_0.006_(PO_4_)_6_(OH)_2_	0.01
Fe-HA3	Ca_9.9625_Fe_0.025_(PO_4_)_6_(OH)_2_	Ca_9.9625_Fe_0.025_(PO_4_)_6_(OH)_2_	0.06
Fe-HA4	Ca_9.925_Fe_0.05_(PO_4_)_6_(OH)_2_	Ca_9.925_Fe_0.05_(PO_4_)_6_(OH)_2_	0.11
Fe-HA5	Ca_9.625_Fe_0.25_(PO_4_)_6_(OH)_2_	Ca_9.665_Fe_0.23_(PO_4_)_6_(OH)_2_	0.52
Fe-HA6	Ca_9.25_Fe_0.5_(PO_4_)_6_(OH)_2_	Ca_9.25_Fe_0.5_(PO_4_)_6_(OH)_2_	1.11
Fe-HA7	Ca_8.5_Fe_1_(PO_4_)_6_(OH)_2_	Ca_8.38_Fe_1.08_(PO_4_)_6_(OH)_2_	2.42

**Table 2:** Powders characteristics of the lattice parameters and crystalline size.

Sample Code	a, nm	c, nm	c\a	V, nm^3^	D, nm
Standard HA (JCPDC #09-0432)	0.9418	0.6884	0.7309	0.5287	-
HA1	0.9467 (7)	0.6898 (6)	0.7286	0.5354	13.8 (0.3)
Fe-HA2	0.9436 (12)	0.6881 (11)	0.7292	0.5306	13.4 (0.3)
Fe-HA3	0.9431 (6)	0.6881 (8)	0.7296	0.5307	13.7 (0.2)
Fe-HA4	0.9437 (2)	0.6881 (2)	0.7291	0.5308	13.2 (0.4)
Fe-HA5	0.9438 (2)	0.6881 (2)	0.7290	0.5308	12.8 (0.4)
Fe-HA6	0.9452 (28)	0.6875 (5)	0.7273	0.5319	10.7 (0.6)
Fe-HA7	0.9442 (18)	0.6875(2)	0.7234	0.5343	9.9 (0.2)

**Table 3:** BET Specific Surface Area, pore volume, and adsorption average pore diameter for the synthesized samples.

Sample	BET Specific Surface Area, m^2^/g	Total Pore Volume, cm^3^/g	BJH Adsorption Average Pore Diameter, nm
HA1	52.1	0.16	16.5
Fe-HA2	85.2	0.18	8.5
Fe-HA3	95.9	0.22	9.5
Fe-HA4	105.7	0.26	9.9
Fe-HA5	134.2	0.28	8.5
Fe-HA6	178.2	0.42	8.9
Fe-HA7	194.5	0.43	8.7

**Table 4:** Parameters of mathematical processing of the Mossbauer spectra of the Fe-HA7 sample obtained at room temperature (isomer shift, IS; quadrupole splitting, QS, hyperfine field, H_hf_; partial area, S_par_).

Sample	Component	IS ±0.01 mm/s	ΔE_Q_ (QS), ±0.01 mm/s	H_hf_, T ± 0.1%	S_par_, ±1%
Fe-HA7	D1	0.37	0.7	-	13
	S1	0.37	−0.2	51.3	87

**Table 5:** The results of in vitro investigations: the pH of the extracts, the values of the optical density (OD) of the formazan solution (MTT test), the pool of viable cells (PVC), and the toxicity index (TI) during the cultivation of the MG-63 cells.

Materials	pH Value of Extract, CGM	MTT Test Results		
		OD, a.u. M (±m)	PVC, %	TI, %
HA1	7.7	0.454 (0.001)	78.5	21.5
HA-Fe5	7.4	0.502 (0.001)	95.8	4.2
HA-Fe7	7.8	0.515 (0.001)	98.3	1.7
Control (CGM)	7.4	0.578 (0.001)	100.0	0.0

## Data Availability

The data presented in this study are available on request from the corresponding author.

## Supplementary Materials

The following are available online at https://www.mdpi.com/2079-4991/11/3/811/s1, Figure S1: XRD spectra of HA and Fe-HA powders after the sintering at 1200 °C, JCPDS# 09-0169 corre-sponds to β-TCP, #09-0432 corresponds to HA.
