# Supplementary material for: Mesoporous Iron(III)-Doped Hydroxyapatite Nanopowders Obtained via Iron Oxalate
Source: Nanomaterials (Basel). 2021 Mar 22;11(3):811. doi: 10.3390/nano11030811 (PMC8005114; doi:10.3390/nano11030811)
Supplement: Supplementary file 1 [file nanomaterials-11-00811-s001.pdf]

Mesoporous Iron(III)-doped Hydroxyapatite Nanopowders Obtained via Iron Oxalate

Margarita A. Goldberg [1,*], Marat R. Gafurov [2,*], Fadis F. Murzakhanov [2], Alexander S. Fomin [1], Olga S. Antonova [1], Dinara R. Khairutdinova [1], Andrew V. Pyataev [2], Olga N. Makshakova [3], Anatoliy A. Konovalov [1], Alexander V. Leonov [4], Suraya A. Akhmedova [5], Irina K. Sviridova [5], Natalia S. Sergeeva [5], Sergey M. Barinov [1] and Vladimir S. Komlev [1]

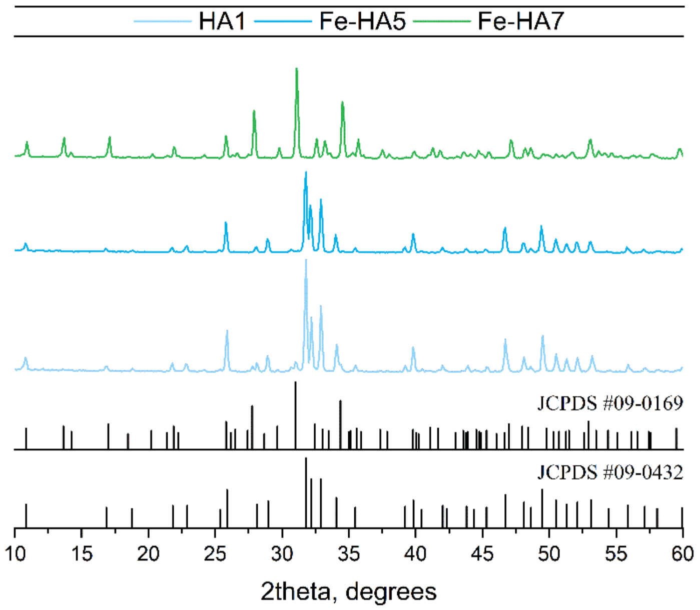

Figure S1. XRD spectra of HA and Fe-HA powders after the sintering at 1200 °C, JCPDS# 09-0169 corresponds to β-TCP, #09-0432 corresponds to HA.
